# Quantitative comparison of the biomass-degrading enzyme repertoires of five filamentous fungi

**DOI:** 10.1038/s41598-020-75217-z

**Published:** 2020-11-20

**Authors:** Magnus Ø. Arntzen, Oskar Bengtsson, Anikó Várnai, Francesco Delogu, Geir Mathiesen, Vincent G. H. Eijsink

**Affiliations:** Faculty of Chemistry, Biotechnology and Food Science, Norwegian University of Life Sciences (NMBU), P.O. Box 5003, 1432 Ås, Norway

**Keywords:** Fungi, Proteomics

## Abstract

The efficiency of microorganisms to degrade lignified plants is of great importance in the Earth’s carbon cycle, but also in industrial biorefinery processes, such as for biofuel production. Here, we present a large-scale proteomics approach to investigate and compare the enzymatic response of five filamentous fungi when grown on five very different substrates: grass (sugarcane bagasse), hardwood (birch), softwood (spruce), cellulose and glucose. The five fungi included the ascomycetes *Aspergillus terreus*, *Trichoderma reesei*, *Myceliophthora thermophila*, *Neurospora crassa* and the white-rot basidiomycete *Phanerochaete chrysosporium*, all expressing a diverse repertoire of enzymes. In this study, we present comparable quantitative protein abundance values across five species and five diverse substrates. The results allow for direct comparison of fungal adaptation to the different substrates, give indications as to the substrate specificity of individual carbohydrate-active enzymes (CAZymes), and reveal proteins of unknown function that are co-expressed with CAZymes. Based on the results, we present a quantitative comparison of 34 lytic polysaccharide monooxygenases (LPMOs), which are crucial enzymes in biomass deconstruction.

## Introduction

Plant biomass, such as woody plants and grasses, are generally called lignocellulose because they are composed of the three main natural polymers: cellulose, hemicellulose and lignin. Lignocellulose is renewable, being both synthesized and degraded by Nature; it is found in forests as well as in agricultural wastes and plays a key role in the Earth’s carbon cycle. It also represents a highly attractive and low-cost feedstock that could be converted into sugars before subsequent fermentation to biogas, bioethanol or other chemicals^1^. Lignocellulose consists of lignin, a heteropolymer of cross-linked polyphenolic units, tightly intertwined with cellulose and hemicellulose, making this material difficult to depolymerize. Still, lignocellulose does not accumulate on Earth due to its removal by the concerted action of highly specialized lignocellulose-degrading microbes.

Saprophytic fungi contribute to carbon recycling by degrading lignocellulose. The genomes of these fungi encode for a vast array of enzymes, including enzymes defined as carbohydrate-active enzymes (CAZymes) that are categorized in the CAZy database^2^. The fungi will produce and secrete a subset of these enzymes that is tailored to the substrate on which they grow^3^. These enzymes include glycoside hydrolases (GHs) that cleave glycosidic bonds, carbohydrate esterases (CEs) that catalyze the deacylation of substituted polysaccharides, and lytic polysaccharide monooxygenases (LPMOs). LPMOs catalyze the oxidative cleavage of glycosidic bonds^4,5^, and have been shown to play an essential role in the degradation of recalcitrant plant polysaccharides, such as cellulose^6^ and xylan^7^. LPMOs have also been show active on starch^8^. In addition to these enzymes, fungi utilize oxidoreductases to modify and depolymerize lignin, including heme peroxidases, laccases, FAD-dependent oxidases and dehydrogenases^9–11^. Of note, these latter enzymes are sometimes also referred to as CAZymes and are part of the CAZy database, where they are categorized as auxiliary activities (AAs)^12^.

A large number of studies focusing on understanding the biomass degrading machinery of fungi have been performed in the past decade. These studies include genome sequencing approaches for identifying the genes potentially involved in biomass degradation for individual fungi^9,13,14^, transcriptomic and proteomic studies for detecting up-regulated genes in response to specific growth conditions and substrates^3,15–17^, and targeted applied studies for characterization of biotechnologically interesting fungal proteins^18,19^. In this study, we present a quantitative comparison of the secretomes of five lignocellulose-degrading filamentous fungi. *Aspergillus terreus* (Ascomycota; Eurotiomycetes; Eurotiales) is a saprophytic ascomycete found in soil and compost and is known to degrade a wide range of plant biomass^20^. *Trichoderma reesei* (Ascomycota; Sordariomycetes; Hypocreales) is a mesophilic ascomycete, famed for degrading textiles during World War II, and is widely used in the enzyme industry due to its high protein secretion capacity^14^. *Myceliophthora thermophila* (Ascomycota; Sordariomycetes; Sordariales), a thermophilic ascomycete, is commonly found in self-heating masses such as compost and is industrially interesting because it degrades wood and plant biomass efficiently and produces thermostable enzymes^21^. *Neurospora crassa* (Ascomycota; Sordariomycetes; Sordariales) is a highly studied ascomycete and (hemi-)cellulolytic model organism found in plants killed by fire^22^. The white-rot basidiomycete *Phanerochaete chrysosporium* (Basidiomycota; Agaricomycetes; Polyporales) secretes a wide range of oxidative and hydrolytic enzymes for degrading lignocellulosic biomass^23^ and is well known for its lignin degrading ability^24^. All five are able to grow on complex lignocellulosic biomass such as sugarcane bagasse, birch wood and spruce wood. Brown-rot fungi were not included in this study as brown-rot fungi are thought to degrade biomass in two stages, i.e. with non-enzymatic followed by enzymatic biomass-degrading machinery^25^, and the method we used here^26^ does not allow for separation of growth phases. We compared expression levels of the CAZymes during growth on these substrates and we report novel, uncharacterized proteins with quantitative expression profiles that suggest their involvement in biomass degradation. Interestingly, the fungi secreted a large repertoire of LPMOs, the individual abundance of which varied significantly between substrates, suggesting considerable functional variation among these important enzymes.

## Results and discussion

### Fungal growth

**Figure 1:**
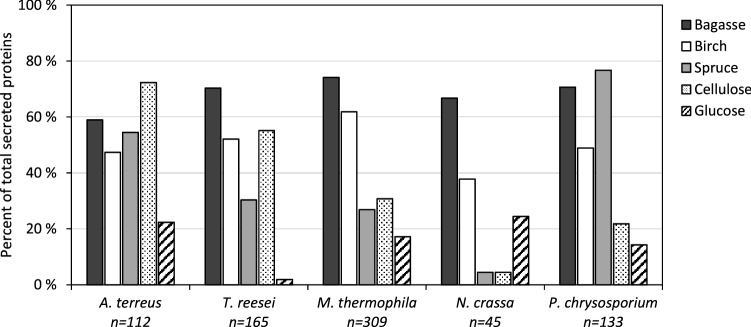
Proteins detected on different substrates. The figure shows the number of identified proteins in secretome samples collected after growth on five different substrates: sugarcane bagasse, birch, spruce, cellulose and glucose. The numbers are calculated as the percentage of the total protein count (denoted as n), i.e., the total number of proteins detected for a fungus when taking data for all five substrates together (see Table 1). The figure was made in Excel from Microsoft 365 (http://microsoft.com).

Five filamentous fungi, well-known for plant cell wall deconstruction, were chosen in this study, four ascomycetes, *A. terreus*, *T. reesei*, *M. thermophila*, *N. crassa*, and one basidiomycete *P. chrysosporium*. Studying secretomes of fungi grown on solid substrates is challenging due to strong adsorption of the secreted proteins to the substrate, as mediated e.g. by carbohydrate-binding modules (CBMs). In many secretome studies, liquid cultures have been used which require relatively harsh sample treatment to recover proteins bound to the substrate, often leading to disruption of cells and contamination of the secretome by intracellular proteins. Here we used a plate-based method for collection of cell-free fungal secretomes developed by Bengtsson et al.^26^. The fungi were grown on agar plates containing one of the five substrates and the secreted proteins were collected from the agar gel below a permeable membrane located beneath the growth site, which serves to filter away cells (illustrated for *T. reesei* in Figure 1 in^26^). This method will arguably minimize contamination by intracellular proteins through unwanted cell lysis, and allow for simple collection of secreted proteins independent of their affinity for the substrate, since also proteins binding to the substrate are collected. On the downside, this method does not allow direct quantification of growth and growth phases, and caution in quantitative interpretation should be exercised as potential side-effects of thermal denaturation on proteins, their interaction with the substrate and (quantitative) diffusion through the membrane onto the sample site are yet to be determined. However, the agar is homogeneous throughout the plate and the sample location is beneath the site of inoculation for all fungi, i.e. the distance of protein diffusion is equal and the protein amounts at the sample location should be comparable.

To assess the ability of the five fungi to adapt their secreted enzyme system according to carbon source, they were grown on three lignocellulosic substrates, grass (sugarcane bagasse), hardwood (birch), and softwood (spruce), as well as on pure (Sigmacell) cellulose and glucose. Key results in terms of the numbers of detected proteins are summarized in Fig. 1 and Table 1 (details are provided in Tables S1–S5 and Figures S1–S5). All five fungi secreted a large number of proteins on all three lignocellulosic substrates, with the exception of *N. crassa* when growing on spruce (Fig. 1). Interestingly, growing the lignin degrader *P. chrysosporium* on cellulose reduced the number of detected proteins to less than half, compared to growth on the lignocellulosic biomass (Fig. 1), and only one lignin-active enzyme (AA2) was detected in the secretome of the cellulose-grown fungus (Table 2, Figure S5). Growing the fungi on glucose, a simple monomeric sugar, yielded lower numbers of detected proteins, with a lesser abundance of polysaccharide-degrading enzymes for all five species (Figures S1–S5). Glucose has been shown earlier to inhibit production of CAZymes in *T. reesei*^27^. Although seemingly low, the numbers of identified proteins (Table 1) are on par with previous literature reporting secretome composition of these fungi using proteomic methods^28–30^, even for *N. crassa* where only 45 proteins were detected^31^. Table 1 shows that the vast majority of the detected proteins were predicted to be secreted, underlining the potential of the plate method in acquiring secretome-enriched samples, although variations in the enrichment for the different fungi were observed (*A. terreus* 91%, *P. chrysosporium* 86%, *T. reesei* 44%, *M. thermophila* 51% and *N. crassa* 56%) indicating that the plate method may not be equally applicable for all organisms. Regardless, only 8–10% of the complete proteomes are predicted to be secreted, indicating a clear enrichment of secreted proteins in the collected secretomes for all sample preparations.

**Table 1:** Protein identification summary for all fungi.

Fungus	Taxonomic rank (phylum; class; order)	Proteins identified	Predicted secreted	CAZymes	Uncharacterized proteins
*Aspergillus terreus*	Ascomycota; Eurotiomycetes; Eurotiales	112	102 (91%)	76 (68%)	19 (17%)
*Trichoderma reesei*	Ascomycota; Sordariomycetes; Hypocreales	165	73 (44%)	54 (33%)	39 (24%)
*Myceliophthora thermophila*	Ascomycota; Sordariomycetes; Sordariales	309	159 (51%)	120 (39%)	101 (33%)
*Neurospora crassa*	Ascomycota; Sordariomycetes; Sordariales	45	25 (56%)	18 (40%)	6 (13%)
*Phanerochaete chrysosporium*	Basidiomycota; Agaricomycetes; Polyporales	133	114 (86%)	95 (71%)	9 (7%)

The table shows the total number of identified proteins in the secretomes for each fungus, based on the joint data obtained for all five substrates. The table also shows the number and fraction of proteins predicted to be secreted, CAZymes and uncharacterized proteins. For each fungus, supplementary material is available. Tables S1–S5 show all proteins identified and their functional annotations, whereas Figures S1–S5 show heat maps of expression levels on the various substrates. The column ‘CAZymes’ includes also stand-alone CBMs, i.e. proteins with no predicted functional domain. The column ‘Uncharacterized proteins’ are proteins annotated as ‘uncharacterized’ by UniProt and proteins for which no peptidase (MEROPS) or carbohydrate-active domains (CAZy) could be predicted.

**Table 2:** Identification of CAZymes in fungal secretomes.

Substrate/enzyme category	Enzyme class	CAZy family	*A. terreus*						*T. reesei*											
			T	Ba	Bi	S	C	G	T	Ba	Bi	S	C	G						
Cellulose	Endoglucanases	GH5, 7, 12, 45	28/11	10	7	9	11	2	13/5	4	2	5	5							
	Cellobiohydrolases	GH6, 7	6/4	4	4	4	4		3/3	2	2	3	3							
	LPMOs	AA9	12/4	3	2	2	4		3/2	2	1	2	2							
	CDHs	AA3_1	1/0						0/0											
	Oligosaccharide oxidases	AA7	34/2	2			2		22/3				3							
Starch	LPMO	AA13	2/1		1		1		0/0											
Xylan	Xylanases	GH10, 11	6/5	5	5	5	5		4/3	2	3	2	2							
	LPMOs	AA14	0/0						1/0											
Arabinan	Endoarabinanases	GH43	20/4	4	2	4	3		3/0											
	Exo-arabinanases/arabinofuranosidases	GH51, 62	6/2	2	2	2	2		1/1			1								
Mannan	Endomannanases	GH5, 26	18/3	3	2	3	3		8/2	2	1	2	2							
Soluble oligosaccharides	β-glucosidases	GH1	3/0						2/1	1	1									
	Mannosidases	GH2	10/0						7/0											
	Xylosidases/β-glucosidases	GH3	21/3	2	2	2	2		13/3	2	3	3	2							
Monosugars	PDHs	AA12	0/0						1/0											
Pectin	Polygalacturonases	GH28	8/1	1		1	1	1	4/1			1								
	Rhamnosidases	GH78	4/0						1/0											
	Pectin lyases	PL1, 3, 4, 20	13/4	4	4	4	3		3/0											
	Pectin methylesterases	CE8	1/0						0/0											
Lignin	Laccases	AA1	7/0						4/0											
	Peroxidases	AA2	3/0						6/0											
	Oxidoreductases	AA3_2-4	15/1				1		9/0											
	Vanillyl-alcohol oxidases	AA4	3/0						4/0											
	Glyoxal oxidases (GLOX)	AA5_1	0/0						1/1	1			1							
	1,4-benzoquinone reductases	AA6	1/0						1/0											
	Noncarbohydrate-active esterases	CE10	65/3	1	3	2	2		30/1				1							
Esterases	Feruloyl/*p*-coumaroyl/acetyl esterases	CE1	17/1	1	1	1	1		12/1	1	1	1	1							
	Acetyl esterases	CE2-5, 12, 16	28/5	5	3	5	4		14/1				1							
	4-*O*-methyl-glucoronoyl esterases	CE15	1/0						1/1	1	1	1	1							
Binding modules	CBM only	CBMx	18/1					1	13/2	2	2	2	2							

Substrate/enzyme category	Enzyme class	CAZy family	*M. thermophila*						*N. crassa*						*P. chrysosporium*					
			T	Ba	Bi	S	C	G	T	Ba	Bi	S	C	G	T	Ba	Bi	S	C	G
Cellulose	Endoglucanases	GH5, 7, 12, 45	16/9	7	6	7	9		14/2	1	2		1		28/9	9	8	9	5	
	Cellobiohydrolases	GH6, 7	8/6	6	6	6	6		8/3	3	3	1	2		8/4	4	4	4	3	1
	LPMOs	AA9	22/12	12	12	11	11		14/5	1	4	1			16/10	9	8	10	3	
	CDHs	AA3_1	2/2	2	2	1	2		2/0						0/0					
	Oligosaccharide oxidases	AA7	16/4	1			3	1	18/0						13/0					
Starch	LPMO	AA13	1/0						1/0						0/0					
Xylan	Xylanases	GH10, 11	10	8	8	4	4		6/3	3	3				7/6	6	6	6	1	
	LPMOs	AA14	0/0						0/0						2/0					
Arabinan	Endoarabinanases	GH43	13/9	9	7	5	2		7/0						4/2	1	2	2		
	Exo-arabinanases/arabinofuranosidases	GH51, 62	4/3	3	1	2	1		1/1	1	1	1			2/1	1	1	1	1	1
Mannan	Endomannanases	GH5, 26	10/4	3	2	3	4		7/1		1				19/5	5	4	5	2	
Soluble oligosaccharides	β-glucosidases	GH1	1/1	1	1				1/0						2/0					
	Mannosidases	GH2	7/2	1	1	1	1		6/0						2/1	1		1		
	Xylosidases/β-glucosidases	GH3	10/2	2	2	2	2		10/1		1				10/3	3	2	3		
Monosugars	PDHs	AA12	3/1	1					3/0						1/1	1				
Pectin	Polygalacturonases	GH28	2/0						2/0						5/4	3	2	4	1	1
	Rhamnosidases	GH78	1/0						1/0						1/1			1		
	Pectin lyases	PL1, 3, 4, 20	8/5	3	4	5	2		4/1	1					0/0					
	Pectin methylesterases	CE8	2/1	1	1	1			1/0						2/2	1	1	2		
Lignin	Laccases	AA1	5/0						10/0						2/0					
	Peroxidases	AA2	4/1	1	1			1	4/0						17/6	5	5	5	1	
	Oxidoreductases	AA3_2-4	5/3	3	2	2	1		2/0						30/1	1	1			
	Vanillyl-alcohol oxidases	AA4	2/0						3/0						1/0					
	Glyoxal oxidases (GLOX)	AA5_1	1/1	1	1	1	1	1	1/0						7/2	2	1	2		
	1,4-benzoquinone reductases	AA6	1/0						1/0						4/0					
	Noncarbohydrate-active esterases	CE10	24/3	2	2	1	1		26/0						35/5	4	4	5		
Esterases	Feruloyl/*p*-coumaroyl/acetyl esterases	CE1	15/4	4	1	2	1		16/0						17/2	2	1	2	1	1
	Acetyl esterases	CE2-5, 12, 16	21/8	7	6	3	2		17/0						18/3	2	1	3		
	4-*O*-methyl-glucoronoyl esterases	CE15	2/1	1	1	1			1/0						2/2	2	2	2		
Binding modules	CBM only	CBMx	14/2	1	1	1	1	1	13/0						32/2	2	2	2	2	

The table shows a subset of CAZymes most relevant for lignocellulose deconstruction and how these are expressed in each fungus, categorized by enzyme class and (expected) substrate of action. In the column ‘T’, the first number shows the total number of predicted CAZymes found in the whole genome using dbCAN while the second number shows the total number of proteins detected in the secretomes across all substrates. The columns labeled Ba: sugarcane bagasse, Bi: birch, S: spruce, C: cellulose, G: glucose, show the number of proteins detected in the secretome during growth on these respective substrates, including if the protein was identified in a single biological replicate, given that it was found in at least two replicates on another carbon source. In two cases (AA3 and AA5), CAZy subfamilies are included for class differentiation. For quantitative expression values, see the heat maps (Figures S1–S5). Note that enzymes listed as active on arabinan may also be active on xylan and pectin due to the presence of arabinosyl groups in these polymers. ‘CBM only’ refers to proteins with no predicted CAZyme function but still containing a carbohydrate-binding module. Note that CAZy enzyme classes may, in some cases, act on more than one substrate. In such cases enzymes are counted as both, e.g. for GH5 which is counted as both endoglucanase and endomannanase.

### Survey of secretome composition across the five fungi

It is clear that the secretome compositions depend on the carbon source (i.e., the substrate), as can be seen in the hierarchically clustered heat maps and in the protein lists for *A. terreus* (Figure S1, Table S1), *T. reesei* (Figure S2, Table S2), *M. thermophila* (Figure S3, Table S3), *N. crassa* (Figure S4, Table S4) and *P. chrysosporium* (Figure S5, Table S5). Overall, we observed that many of the detected proteins have similar expression patterns for all three lignocellulosic substrates. On the other hand, the fungi do show differences. For example, for *A. terreus*, the response is highly similar for all three substrates, but the other four fungi show considerable numbers of substrate specific proteins. Interestingly, all fungi, except *N. crassa*, express multiple proteins unique to growth on cellulose and many of these are uncharacterized.

**Figure 2:**
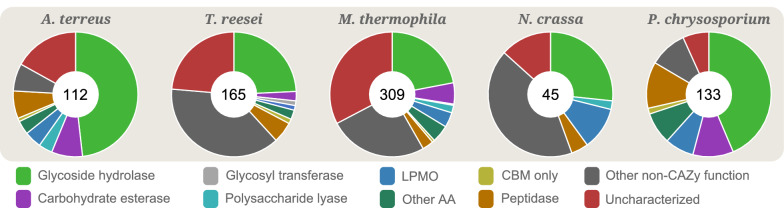
Functional annotation of the secretomes of all five fungi. The figure shows the proportion of CAZymes in the secretomes for all the five fungi, irrespective of substrate used for growth. The number in the center indicates how many proteins that were detected in the secretome of each fungus. LPMO: Lytic polysaccharide monooxygenase. AA: auxiliary activity, i.e. redox enzymes that work in conjunction with CAZymes. CBM: carbohydrate binding module. ‘CBM only’ refers to proteins where no CAZyme function could be predicted, yet they contain a CBM (see main text for details). ‘Uncharacterized’ refers to proteins annotated as such by UniProt and where no peptidase or carbohydrate-active domains could be predicted by MEROPS or dbCAN, respectively. Proteins predicted as both carbohydrate esterases and as peptidases, by dbCAN and MEROPS, respectively, are counted just as carbohydrate esterases (see main text for details). The figure was made in Excel from Microsoft 365 (http://microsoft.com) and Inkscape v0.48.4 (https://inkscape.org/).

**Figure 3:**
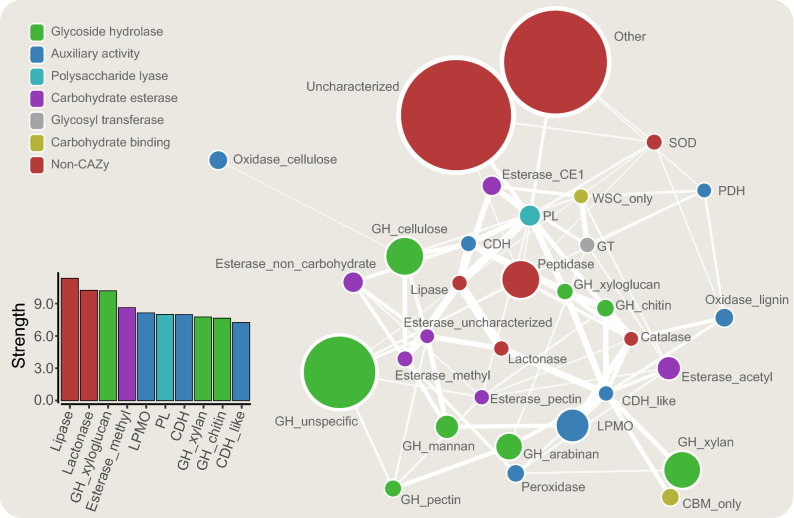
Co-expression network. The figure shows a summarized interaction network of enzyme classes found in the five fungal secretomes. The size of the nodes reflects the number of proteins in each class, whereas the edges (lines) and their thickness reflect the degree of co-expression. The visualization was generated with a layout to reflect the node strength (spring-embedded), meaning that enzyme classes with high co-expression are close to one another. Note that while the network shows how likely enzymes from two classes are to be expressed (and secreted) together, it may overlook singular meaningful cooperation cases, e.g. a single enzyme expressed only on one specific substrate. Only significant edges are shown (marginal likelihood filter p < 0.05). For each node, we computed the sum of all the weighted edges connected to it (strength). The strength score (bottom left panel) reflects how much the enzymes from a certain class are expressed together with enzymes of other classes and is used for detection of hub classes. For enzyme class abbreviations, see text. The figure was made with R v3.6.1 and Cytoscape v3.7.1.

As fungi usually degrade polysaccharides outside the cell and import the generated oligo- or monosaccharides for further intracellular metabolism, a large fraction of the secretomes is expected to be CAZymes. We found that, on average, 50% of the detected proteins in the secretomes, in fact, were CAZymes (Table 1; in total 363 CAZymes), and that many of those that were not were uncharacterized proteins, i.e. proteins that could potentially be involved in substrate degradation or metabolism, although with a hitherto unknown substrate affinity and function. Glycoside hydrolases (GHs) were the most prevalent family, constituting close to half of the proteins detected in the secretome of *A. terreus* and *P. chrysosporium*, and a fourth of the proteins detected for the other fungi (Fig. 2). In total, 232 GHs were identified encompassing 51 GH families, the most prevalent being GH5, GH10 and GH43 (15 proteins each), GH7 (13 proteins), GH3 (12 proteins), GH11 (10 proteins), GH16 (9 proteins), GH30 (8 proteins), GH6, GH12, GH18, GH31, GH55 and GH72 (7 proteins each). In addition to GHs, we detected 44 carbohydrate esterases (CEs; 43 predicted to be secreted). These enzymes catalyze the deacylation of substituted polysaccharides, such as the deacetylation of xylan, as found in birch and sugarcane bagasse, or of glucomannan, as found in spruce and birch. Moreover, 11 polysaccharide lyases (PL; 10 predicted to be secreted) were identified. As many as 67 of the detected CAZymes were auxiliary activity (AA) proteins, including lignin-degrading redox enzymes and lytic polysaccharide monooxygenases (LPMOs). In total, we detected 34 LPMOs (33 AA9s and one AA13), showing diverse expression between the substrates (see below), seven AA2s, eight AA3s, four AA5s, nine AA7s, three AA8s and two AA12s. The CAZymes, in general, showed a high level of co-expression (Fig. 3; closely connected with thick edges forming the core of the network), while uncharacterized proteins and proteins with a known non-carbohydrate active function were located more distantly in the network representation (Fig. 3; red).

In total, we detected seven proteins with no predicted CAZyme function containing a carbohydrate-binding module (CBM) (Table 2; *A. terreus*: Q0CV89; *T. reesei*: G0R6T7, G0RVK3; *M. thermophila*: G2QCL0, G2QI84; *P. chrysosporium*: 2762164616, 2762165332). Two of these were only expressed on glucose (Q0CV89, G2QCL0), while the other five were expressed on all substrates except glucose. Using MEROPS we could identify a peptidase domain in 2762164616, while searches in InterPro and Pfam did not reveal additional putative functions for any of these proteins, except for G0RVK3, which contains an expansin domain in addition to a CBM1. Expansins are plant cell wall proteins involved in cell wall loosening and play a key role in plant cell growth, while they are also found in plant biomass-degrading fungi and bacteria, such as in *Clostridium clariflavum*, where expansins are incorporated into cellulosomes^32^. Expansins are thought to break non-covalent bonds between cell wall polysaccharides and have been shown to facilitate degradation of cellulosic biomass by cellulases^32,33^.

The data further show that the fungal response to growth on lignocellulose includes induction of peptidases predicted to be secreted. Although this has been reported previously^20^, the exact role of peptidases in lignocellulose degradation is not understood. It has been speculated that peptidases could degrade plant cell wall proteins, thereby giving increased access for GHs to degrade polysaccharides^34^. We detected in total 57 proteins that were predicted as peptidases according to MEROPS (Tables S1–S5); however, 13 of these contained also a CAZy domain, mostly CE, indicating that these enzymes could primarily function as esterases. Of the remaining 44 peptidases, 29 were expressed during growth on polysaccharides only (i.e., not on glucose). Four of these were cellulose specific (Q0CVY4, Q0CAE1, Q0C8H2, G0R8T0), while the rest were either expressed on all lignocellulosic substrates or were specific to one of them. They showed overall weak co-expression with other carbohydrate-active enzymes (Fig. 3; thin edges). More studies to elucidate the role of peptidases in lignocellulose deconstruction are of interest.

### A closer look at proteins with no annotated carbohydrate-active function

**Figure 4:**
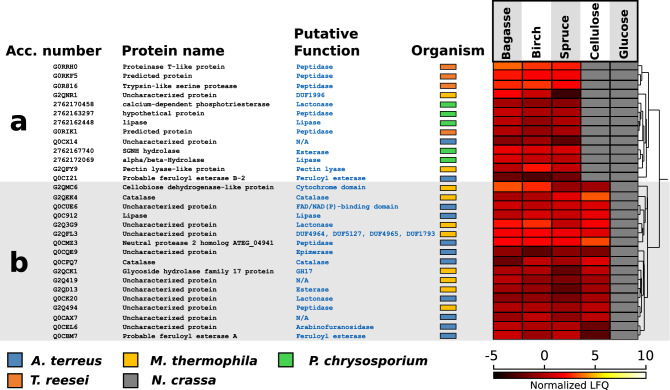
Heat map representations of selected non-CAZy proteins. The figure shows the expression patterns of non-CAZy proteins that are predicted to be secreted and that are highly expressed when grown on (**a**) lignocellulose and (**b**) polysaccharides. Every row in the heat map represents a protein, and the colors represent the protein abundance (average of three replicates) when grown on five different substrates: sugarcane bagasse, birch, spruce, cellulose and glucose. The scale of the heat map ranges from high abundance (white color, normalized log2-based LFQ at 10) to low abundance (black color, normalized log2-based LFQ at − 5). The grey color indicates that the protein was not detected. Putative functions were assigned using Pfam and InterPro. Non-CAZy proteins with more diverse expression patterns, can be found in Figure S6. The figure was made with Perseus v1.6.0.7 and Inkscape v0.48.4 (https://inkscape.org/).

As with all large-scale proteomics studies, we detected several abundantly expressed “unknowns”, here defined as proteins with no annotated carbohydrate-active function (yet a different functional annotation in UniProt or Pfam may exist). Of these proteins, those that are predicted to be secreted and highly expressed during growth on lignocellulose (and possibly also on cellulose but not on glucose) may be involved in biomass conversion. When analyzing for this, 30 proteins stood out as interesting due to their abundant and consistent expression when the fungi grew on lignocellulosic substrates, but not on cellulose (Fig. 4a) or on all polymeric substrates (both lignocellulose and cellulose; Fig. 4b). When using Pfam and InterPro to allocate putative functions for these, we detected two potential feruloyl esterases from *A. terreus* (Q0CBM7, Q0CI21). Feruloyl esterases have previously been shown to act synergistically with xylanases, cellulases and pectinases in breaking down complex cell wall carbohydrates^35,36^, and some feruloyl esterases are able to cleave next to diferulic bridges that crosslink layers of hemicellulose in the plant cell wall^37^. In addition, we identified three proteins as ‘lipase’ (2762162448 and 2762172069 from *P. chrysosporium*, and Q0C912 from *A. terreus*), which in a multiple sequence analysis clustered together with the feruloyl esterase Q0CBM7 from *A. terreus* (data not known). Interestingly, feruloyl esterases contain an alpha/beta hydrolase fold typical of lipases (InterPro: Fungal lipase-like domain; IPR002921). It is possible that these lipases may also contribute to deacylation of the plant cell wall, and the fact that they are observed in the secretome of two different fungi is interesting. Notably, previously annotated CE1 esterases (feruloyl/*p*-coumaroyl/acetyl) and lipases show relatively strong co-expression (Fig. 3). Additionally, two other potential esterases were detected among the proteins in Fig. 4 (2762167740, G2QD13).

Figure 4B contains a protein referred to as cellobiose dehydrogenase (CDH)-like protein (G2QMC6), comprising only the cytochrome domain of CDH. Cytochrome domains can transfer electrons between redox enzymes and could play a role in activating LPMOs by acting as an electron shuttle. In some cases, we detected CDH-like enzymes which lacked either the cytochrome or the FAD domain found in CDHs. These proteins showed strong co-expression with LPMOs (Fig. 3), suggesting some sort of auxiliary enzyme function remaining, despite the lack of a complete CDH architecture. Another possibility is that they are regulated under a similar promoter. Certainly, more research on elucidating the function of CDH-like enzymes is of interest. Figure 4 also contains three putative lactonases (2762170458, Q0CK20, G2Q3G9). The latter, from *M. thermophila,* has been previously characterized as an extracellular aldonolactonase and postulated to work in synergy with CDHs or glucose oxidases^18^. Lactonases catalyze the hydrolysis of sugar lactones generated by e.g. CDH or LPMOs, to aldonic acids. Sugar lactones inhibit glycoside hydrolases^19^, and their removal may thus be beneficial during biomass conversion. On the other hand, low concentrations of cellobionolactone have been shown to induce a fungal response tailored for degrading crystalline cellulose in *T. reesei*^38^. Lactonases are found in nearly all known cellulolytic ascomycetes, and it is interesting to observe that this enzyme class is being expressed only during growth on polymeric substrates, and by three different species. In the co-expression network (Fig. 3), they form a hub enzyme class (high node strength); perhaps in fact lactonases are key regulators of cellulase activity working to keep lactone concentrations low but high enough to induce cellulase expression^39^.

Two proteins in Fig. 4 contained domains of unknown functions (DUFs). G2QNR1 contained DUF1996 (Pfam: PF09362), a domain found to be numerically elevated in *M. thermophila*, *C. globosum, N. crassa* and *T. terrestris* compared to other Chaetomiaceae fungi^13^. DUF1996 domains often occur together with CBM (CBM1 or CBM6) or WSC domains (Pfam: PF01822). WSC domains are potential carbohydrate binding modules previously found in fungal extracellular exoglucanases^40^. Thus, DUF1996 may be associated with polysaccharide degradation. The other protein, G2QFL3, had four unknown domains, DUF4964, DUF5127, DUF4965 and DUF1793 (Pfam: PF16334, PF17168, PF16335 and PF08760, respectively), where two of the domains seem to be part of a GH superfamily according to InterPro, suggesting that these DUFs may, potentially, be glycoside hydrolases.

We detected seven additional peptidases among the previously non-annotated proteins in Fig. 4 (G0R816, G0RIK1, G0RKF5, G0RRH0, Q0CMZ3, 2762163297 and G2Q494), as well as two catalases (Q0CFQ7 and G2QEK4), one arabinofuranosidase (Q0CEL6), one GH17 (G2QCK1) not recognized by dbCAN, a protein containing a pectin lyase fold (G2QFY9), a possible epimerase (Q0CQE9), and one protein containing a single FAD/NAD(P)-binding domain (Q0CUE6). For the final three proteins, no functional annotation could be predicted (Q0CX14, Q0CAX7 and G2Q419).

In addition to the 30 proteins in Fig. 4, we identified 70 proteins with a more diverse expression pattern which separated into three clusters where cluster I and III contained proteins showing different expression patterns for the three different lignocellulosic substrates, i.e. inconsistent or possibly substrate dependent, and cluster II contained cellulose-specific proteins. For these 70 proteins, we did not perform an in-depth functional analysis like for the 30, but they are included in Figure S6 for future reference. Taken together, quantitative proteomics to correlate expression patterns, both with heat maps (Fig. 4 and Figure S6) and co-expression network analysis (Fig. 3), constitutes a powerful method for detecting non-CAZy proteins potentially involved in lignocellulose degradation.

### The fungal secretomes are adapted to the substrates

To utilize complex substrates as nutrients, fungi up-regulate genes encoding specific enzymes for targeted deconstruction of such substrates^41^. The three lignocellulosic substrates used in this study (grass, hardwood and softwood) differ in their general composition. While grasses, such as sugarcane bagasse, are rich in pectin, xyloglucan, xylan and mixed-linkage glucans, the main hemicelluloses in woody materials, such as birch and spruce, are xylan and glucomannan. In addition, grass, hardwood and softwood xylans and glucomannans differ in their substituting groups (acetylations and branching sugars).

Overall, the fungi showed fairly similar CAZyme profiles when grown on all four polymeric substrates, in terms of both enzymatic repertoire (Table 2) and expression levels (Figures S1–S5), while CAZymes were less expressed on glucose. The low CAZyme expression by glucose is well known from previous studies^27^. For *A. terreus*, we observed close-to-identical secretomes for the three lignocellulosic substrates (Figure S1), indicating that a broad subset of CAZymes is co-regulated in this fungus. The other four fungi showed larger substrate-dependent variations in their secretomes, suggesting a higher level of substrate-dependent adaptation. For *T. reesei*, the total number of proteins detected on sugarcane bagasse was more than two times that for spruce (Fig. 1), but when considering only the most abundant proteins, the response to all three lignocellulosic substrates was quite similar (Figure S2). *M. thermophila* is known to hydrolyze all major polysaccharides found in biomass^13^, and this was reflected in a massive secretome response, both in terms of total protein count (Table 1) and CAZyme-count (Table 2). The latter is especially true for hemicellulose-rich bagasse and birch, whereas the number of detected proteins was considerably lower for spruce (Figure S3), which is poorer in hemicellulose due to the pretreatment procedure that was used. Compared to the other fungi and in terms of protein count, the response of *N. crassa* was limited and showed large differences between the substrates (Figure S4). Only few proteins were detected in the spruce and cellulose samples, whereas a much richer secretome was detected for (the hemicellulose-rich) bagasse and birch. The white-rot fungus *P. chrysosporium* secreted a broader range of CAZymes when grown on the three lignocellulosic substrates than when grown on pure cellulose (Figure S5). In this case, most proteins were detected in the spruce samples, underpinning the varying effects of the substrate on fungal secretomes. Although *P. chrysosporium* is known for its ability to degrade lignin efficiently, as it indeed secreted a few peroxidases at high abundance during growth on the three lignocellulosic substrates (Figure S5; 2762171893 and 2762168649), its secretome contained a broad range of CAZymes. In general, among the most abundant proteins for all the fungi, we found several enzymes harboring a CBM1, a carbohydrate-binding module known to bind crystalline cellulose.

Cellulose is composed of β-1,4-linked glucose units and is present in all four polymeric substrates. Accordingly, the secretomes of all fungi grown on these substrates showed a combination of β-glucosidases (GH1, GH3), cellobiohydrolases (GH6, GH7), endoglucanases (GH5, GH7, GH12, GH45) and LPMOs (AA9) (Table 2). Other detected enzymes relevant for cellulose degradation include CDH (*M. thermophila*), PDH (*M. thermophila* and the white-rot basidiomycete *P. chrysosporium*) and various putative glucooligosaccharide oxidases (AA7; two for *A. terreus* on sugarcane bagasse and cellulose; three for *T. reesei*, all exclusively expressed during growth on cellulose; three for *M. thermophila* primarily expressed on cellulose). Interestingly, two of the AA7s are expressed with high abundance (G0R6T3 in *T. reesei* and Q0CF74 in *A. terreus*).

Hemicellulose consists of several heteropolymers. Xylan occurs in sugarcane bagasse as acetylated glucuronoarabinoxylan, in birch as acetylated methylglucuronoxylan, and in spruce as arabinoglucuroxylan. Glucomannan is found in woody plant cell walls and, unlike in birch, glucomannan in spruce is galactosylated and acetylated. Detected hemicellulases include xylosidases (GH3, GH43), xylanases (GH10, GH11), xyloglucanase (GH74), fucosidase (GH29), arabinofuranosidases (GH43, GH51, GH54, GH62), galactosidase (GH27), mannosidases (GH2) and endomannanases (GH5, GH26) (Table 2). For some fungi hemicellulases were less abundant in the cellulose samples, compared to lignocellulose samples, but this expected trend varied in strength between the fungi, being strong for *P. chrysosporium* and absent for *A. terreus*. One GH5 was strongly expressed by *T. reesei* on spruce and cellulose, but in much lower levels on sugarcane bagasse and birch (G0RC85). This GH5 belongs to the subfamily 7, which contains characterized mannanases. Similarly, three GH5 subfamily 7 enzymes were detected in the secretome of *P. chrysosporium*, where two of them showed the highest expression on spruce (2762167022, 2762171758). This is a clear adaptation of fungal response to substrate composition, as spruce contains more structural mannan than the other lignocellulosic substrates. Recently, a new LPMO was reported, seemingly active on xylan, and this has been placed in a new AA class, AA14^7^. Both *T. reesei* and *P. chrysosporium* code for AA14 enzymes, yet none of these were detected in the secretomes (Table 2). PDHs (AA12), detected for *M. thermophila* and *P. chrysosporium* growing on sugarcane bagasse, may also be active on hemicellulosic sugars.

As expected, multiple esterases were detected, primarily during growth on hemicellulose-rich substrates, including various acetylesterases (CE2, CE3, CE4, CE5, CE12, CE16), feruloyl and *p*-coumaroyl esterases (CE1), and methylesterases, both pectin-active (CE8) and 4-*O*-methyl-glucuronoyl-active (CE15). *A. terreus* secreted nine CEs where two of these, a CE5 acetylxylan esterase (Q0CNM5) and an uncharacterized CE1 esterase (Q0CWM0), contained a CBM1 and were highly expressed on all polymeric substrates. For *T. reesei*, four CEs were detected, and two of these were highly abundant: one CE5 esterase (G0R6T6; with CBM1) expressed only on pure cellulose and one CE15 (discussed below). *M. thermophila* secreted four CE1 esterases, two of them being sugarcane bagasse-specific (G2Q8Y7, G2QMB4), as well as four CE3 acetyl esterases, one sugarcane bagasse-specific, one spruce-specific, and two in common for sugarcane bagasse and birch. With the exception of the CE16 acetylesterase (G2QJ27), all *M. thermophila* esterases had lower abundance on spruce than on sugarcane bagasse and birch. No esterases were detected in the secretome of *N. crassa* although its genome codes for several of them. Esterases detected for *P. chrysosporium* included two CE1 esterases, where one was expressed on all substrates including glucose, one CE2 and two CE16s. Interestingly, one of the CE1 esterases was expressed in very high abundance on all polymeric substrates (2762170282). Sugarcane bagasse and birch contain methylglucuronoxylan, and 4-*O*-methyl-glucoronoyl methylesterases (CE15), enzymes that targets the substituting groups of this polymer, were indeed detected. *T. reesei* expressed one CE15 with a CBM1 in large amounts on all polymeric substrates, while the CE15 from *M. thermophila* was only expressed on sugarcane bagasse and birch. *P. chrysosporium* expressed two CE15s on all lignocellulosic substrates, one with high (with a CBM1; 2762171709) and one with low abundance.

Pectins are a heterologous group of large and complex acidic polysaccharides mainly made up of partly methylated polygalacturonic acid and a rhamnose-galacturonic acid copolymer substituted with arabinogalactan side chains. Pectin-active enzymes were detected for all five fungi, but with large variations. While the *A. terreus* secretomes contained one polygalacturonase (GH28) and four pectin lyases (two PL1s, one PL3 and one PL4), only one GH28 polygalacturonase was found secreted by *T. reesei*, and only on spruce and in low amounts. The *M. thermophila* genome codes for eight pectin lyases, and two of these (one PL1 and one PL3) were strongly expressed on birch and in lesser amounts on the other substrates. *M. thermophila* also expressed one pectin esterase (CE8) on lignocellulosic substrates. The secretomes of *P. chrysosporium* contained four GH28 polygalacturonases, one GH78 rhamnosidase and two CE8 pectin esterases. Interestingly, no pectin lyases can be found in the genome of *P. chrysosporium*.

In addition to the abovementioned CAZymes, members of less studied classes, such as various GHs (GH16, GH18, GH28, GH31, GH35, GH79, GH93, GH115, GH131) were detected for some of the fungi on some of the substrates (see Tables S1–S5 for details). Moreover, we detected 12 CE10s, which are believed not to be active on carbohydrate substrates. Since these enzymes were never expressed when the fungi were grown on glucose, it is possible that these enzymes do play a role in biomass conversion, perhaps related to modifying lignin. Three *A. terreus* CE10s were detected: Q0CUG8 was present in all secretomes with polymeric substrates, Q0C8V9 was found with woody substrates but not with sugarcane bagasse, and Q0CZB3 was only expressed on birch. For *T. reesei*, only one low abundant CE10 (G0RFR3) was detected and only on the cellulosic model substrate. The secretomes of *M. thermophila* contained three CE10s, where one (G2QFS5) was highly expressed on sugarcane bagasse and birch, and much less expressed on spruce. *P. chrysosporium* expressed the highest number of CE10 esterases: four were detected with the three lignocellulosic substrates at varying levels, and a fifth one (2762166294) was only detected with spruce. The secretomes of the three Ascomycetes, *A. terreus*, *T. reesei* and *M. thermophila*, and of the known lignin-degrading Basidiomycota *P. chrysosporium* also contained lignin-degrading enzymes. These enzymes will be discussed in detail below.

### LPMOs seem to play a crucial role in substrate degradation

Lytic polysaccharide monooxygenases (LPMOs) cleave glycosidic linkages in cellulose and, in some cases, other β-glucans by oxidizing either the C-1 or the C-4 carbon in the presence of molecular oxygen or hydrogen peroxide^4,7,11,42–44^. LPMOs play an instrumental role in deconstruction of recalcitrant crystalline biomass^45,46^ and have been the subject of study from several fungal secretomes using proteomics, including *Aspergillus nidulans*^16^, *T. reesei*^26^*, Aspergillus aculeatus*^47^*, Phlebia brevispora* and *Bjerkandera adusta*^48^, and even in the early-lineage zoosporic fungus *Rhizophlyctis rosea*^49^. Their central role is also evident from the co-expression network representation, where LPMOs stand out as being one of the hub enzyme classes (Fig. 3; high node strength). Importantly, recent work shows that LPMOs may require H_2_O_2_ to function, and that control of H_2_O_2_ levels is important to avoid autocatalytic LPMO inactivation^5,11,43,50–52^. When looking at their closest connections, i.e. enzyme classes showing significant co-regulation (thick edges), we observe CDH-like enzymes, enzymes that are able to produce H_2_O_2_, and catalases, enzymes that function to control H_2_O_2_ levels. It is tempting to speculate whether there might be some interaction between the H_2_O_2_ producers (CDHs, PDH, oxidases and superoxide dismutase) and H_2_O_2_ consumers (LPMOs, peroxidases and catalases) given the connections between these nodes in the co-expression network and that the preferred co-substrate for LPMOs is likely to be H_2_O_2_^43,50^.

**Figure 5:**
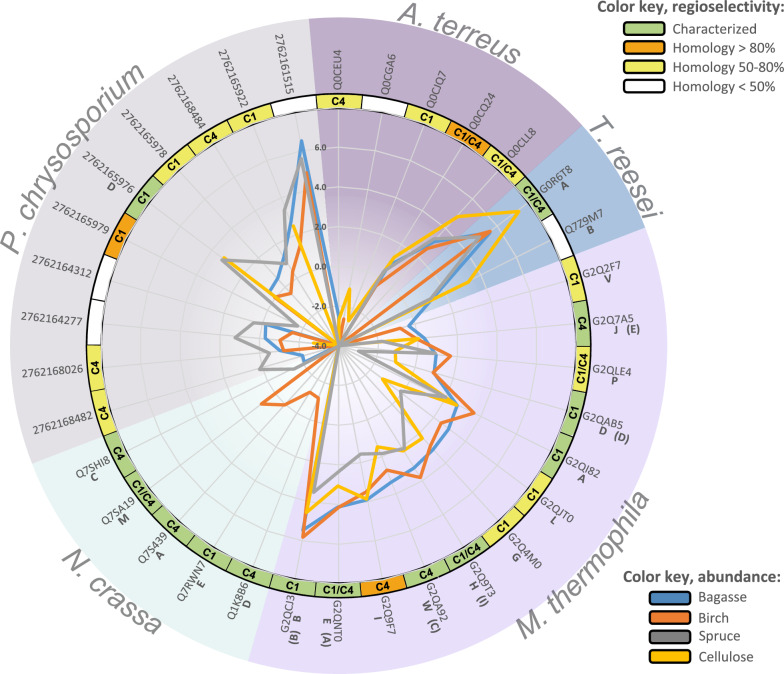
LPMO expression profiles. The figure shows expression levels for all detected LPMOs for all five fungi grown on sugarcane bagasse, birch, spruce or cellulose. Glucose was left out as no LPMOs were expressed during growth on that substrate. The scale refers to log2-based and normalized LFQ values (the same as those shown in the heat maps of Figures S1–S5) and ranges from − 4.0 (center of circle) to 8.0. All LPMOs belong to the AA9 family, except for Q0CGA6 (*A. terreus*), which is an AA13. LPMOs that have previously been characterized (see text for references) are marked with green while LPMOs that share high sequence identity with characterized LPMOs are indicated by an orange (> 80%) or yellow (> 50%) color. For all LPMOs marked as previously characterized (green), activity on cellulose has been demonstrated. The known or predicted position of hydroxylation during oxidative cleavage is indicated as C1, C4 or C1/C4. LPMO letters are based on nomenclature from Berka et al.^13^ and Kadowaki et al.^56^ , while letters in brackets are from Frommhagen et al.^81^. The figure was made with Excel from Microsoft 365 (http://microsoft.com) and Inkscape v0.48.4 (https://inkscape.org/).

The secretome dataset contained quantitative data of 34 expressed LPMOs from five fungi growing on four different substrates, 33 belonging to AA9 and one belonging to AA13 (Fig. 5; no LPMOs were detected during growth on glucose). The genomes of *T. reesei* and *P. chrysosporium* also encode for AA14s (xylan-active LPMOs), but none were expressed (Table 2). Regarding chitin-active LPMOs (AA11^53^) and the new class AA16^47^, some were found encoded in the genomes but none could be detected in the secretomes, suggesting that they are not expressed under these conditions. AA15 is thought not to be a fungal enzyme, and as expected, no AA15 could be found in the genomes of the five fungi.

Five LPMOs (including the one AA13) were detected for *A. terreus* but only two of these, both AA9, showed high abundance (Q0CQ24 and Q0CLL8). These two are predicted to possess both C1- and C4-oxidizing activity, based on sequence similarity with functionally characterized LPMOs, and are expressed on all three lignocellulosic substrates and, to a slightly higher extent, on pure cellulose. *T. reesei* expressed two LPMOs, *Hj*LPMO9A (G0R6T8) and *Hj*LPMO9B (Q7Z9M7), where *Hj*LPMO9A showed higher abundance and was expressed on all four substrates. This is a previously characterized LPMO with C1/C4-oxidizing activity^54,55^. *Hj*LPMO9B was expressed with a lower abundance overall and not expressed at all during growth on birch. *T. reesei* is a well-known cellulose degrader, and the observation that the expression levels of these LPMOs are highest for cellulose is therefore not surprising. For *M. thermophila*, as many as 12 LPMOs were detected. While all of these are expressed during growth on sugarcane bagasse and birch, several, *Mt*LPMO9L (G2QJT0), *Mt*LPMO9P (G2QLE4), *Mt*LPMO9J (G2Q7A5) and *Mt*LPMO9D (G2QAB5) show clearly lower levels or were absent (*Mt*LPMO9V; G2Q2F7) during growth on spruce and cellulose (nomenclature according to Berka et al.^13^ and Kadowaki et al.^56^). *Mt*LPMO9D, previously characterized as a C1-oxidizing LPMO^57^, showed the highest expression levels during growth on sugarcane bagasse and birch. It is worth noting that the genome of *M. thermophila* encodes 22 AA9 LPMOs, and that 10 of these remained undetected in the secretome despite the variations in substrate. Thus, while there clearly are substrate-dependent variations in expression levels for individual LPMOs, overall, the same subset of twelve LPMOs is used. This is corroborated in the study by Berka et al. where the secretome of *M. thermophila* when growing on alfalfa and barley straw showed expression of eleven LPMOs^13^, all overlapping with our dataset. In addition, we identified a twelfth, *Mt*LPMO9V. The *N. crassa* secretomes contained five LPMOs, but at low levels compared to the other fungi. Four of them were only found with birch: *Nc*LPMO9A (Q7S439, C4-oxidizing^58^), *Nc*LPMO9D (Q1K8B6, C4-oxidizing^58^), *Nc*LPMO9E (Q7RWN7, C1-oxidizing^58^ ), and *Nc*LPMO9M (Q7SA19, C1/C4-oxidizing^58^). On the other hand, *Nc*LPMO9C (Q7SHI8, C4-oxidizing^59^) was only detected with spruce and sugarcane bagasse. *P. chrysosporium* samples showed 10 LPMOs, of which only three were expressed during growth on cellulose (2762165976, 2762165922 and 2762164277) and one seemed to be spruce-specific (2762165979). The others were detected with all three lignocellulosic substrates. Interestingly, the most abundant *P. chrysosporium* LPMO, 2762161515, shows less than 50% sequence identity with all functionally characterized LPMOs, making this LPMO an interesting target for further research.

Comparing the abundances of the LPMOs to the rest of the expressed proteins, it is evident that some of the LPMOs are amongst the highest expressed proteins (normalized LFQ values > 2) in the secretomes, across all substrates, underlining that these enzymes play a crucial role in substrate degradation. The LPMO expression data show substrate-dependent variations in expression levels of individual LPMOs and point at LPMOs with potentially interesting highly specific functions that warrant further research. Examples of such LPMOs are 2762168484 and 2762161515 from *P. chrysosporium* that were abundantly detected in samples from sugarcane bagasse, birch and spruce only, and Q7Z9M7 from *T. reesei* that was abundantly detected from sugarcane bagasse, spruce and cellulose, while not expressed on birch.

### Deconstruction of lignin

Lignin constitutes 20–30% of woody plant cell walls and is an aromatic heteropolymer built up by phenylpropanoid subunits linked together with various ether and carbon–carbon bonds. It is tightly interlaced with hemicelluloses covering the cellulose microfibrils and therefore forms a barrier to polysaccharide degradation by CAZymes. Hence, plant cell wall-degrading fungi have developed a set of enzymes to modify or remove lignin. Ascomycetes are thought to mainly degrade carbohydrates in their natural environment^60^. In accordance with this, very few and low abundant lignin-modifying enzymes could be detected in their secretomes (Table 2). *A. terreus* expressed one AA3 subfamily 2 oxidoreductase (Q0CFL8) on cellulose. *T. reesei* expressed one glyoxal oxidase (GLOX; AA5 subfamily 1) (G0RXI7) on sugarcane bagasse and cellulose. *M. thermophila* expressed one AA2 peroxidase (G2QBT1) on sugarcane bagasse, birch and glucose, one GLOX (G2Q335) on all substrates (including glucose), and three AA3 subfamily 2 oxidoreductases (G2PZJ2, G2QKM4 and G2QDZ2) on all polysaccharide substrates, on sugarcane bagasse and birch and on sugarcane bagasse, respectively.

The genomes of these three Ascomycetes code for several laccases (AA1; Table 2), suggesting that they, although not reflected in the secretomes, may possess enzymatic machinery to modify lignin. However, not all laccases are involved in ligninolysis but may have other functions, e.g. in morphogenesis and stress defense^61^. The lack of laccases in Ascomycete secretomes is well known from work on *M. thermophila*, which showed a lack of laccases during growth on various substrates, including barley, oat, triticale, alfalfa, canola, glucose, flax and wood and corn stover pulps^13,41^. The reason why these Ascomycetes do not up-regulate laccase production during growth on lignocellulose under laboratory conditions remains unknown. Upregulation may require an inducer such as 2,5-xylidine^62^, or laccase expression may be hampered by environmental conditions such as pH, temperature or time of secretome collection.

Basidiomycetous white-rot fungi are capable of metabolizing lignin using a number of oxidoreductases^63^, and several such oxidoreductases were indeed detected in the secretomes of *P. chrysosporium* (Table 2 and Figure S5). The genome of *P. chrysosporium* encodes ten lignin peroxidases (LiPs; targeting non-phenolic parts of lignin), five manganese peroxidases (MnPs; targeting phenolic parts of lignin) and one versatile peroxidase (VP; targeting both aliphatic and phenolic lignin structures)^9,23^. Of these AA2 enzymes, we found five that were abundantly and exclusively expressed during growth on lignocellulose (2762170727, 2762171888, 2762166069, 2762168649 and 2762171893), while one (2762167606) was expressed specifically and less abundantly on cellulose (Table 2 and Figure S5). It is possible that the latter enzyme, unlike the other AA2s, shares a regulatory pathway with cellulose-active enzymes. AA2 peroxidases require extracellular H_2_O_2_ for their catalytic activity and the likely source have, according to previous studies, been attributed to copper radical oxidases and alcohol oxidases^9^. Accordingly, we found two copper radical oxidases in the secretome of *P. chrysosporium*: glyoxal oxidases (GLOX; AA5 subfamily 1) 2762169585 and 2762165280. The latter, which has been previously characterized^64^, was highly expressed during growth on sugarcane bagasse and birch, less abundantly expressed on spruce and not expressed at all on pure cellulose or glucose (Figure S5). The other GLOX (2762169585) was expressed in low amounts on sugarcane bagasse and, to an even lesser extent, on spruce. Interestingly, LiPs are able to produce one of the substrates for GLOX, glycolaldehyde, by Cα-Cβ-cleavage of arylglycerol-β-aryl ethers in lignin. Oxidation of glycolaldehyde by GLOX to oxalic acid may lead to the generation of three molecules of H_2_O_2_^65^. In addition to these two GLOXs, H_2_O_2_ may also be produced by the AA12 dehydrogenase 2762169982, the cellobiose dehydrogenase 2762169415 and the alcohol oxidase 2762166594, all three detected in the secretomes of *P. chrysosporium*. Such enzymes have been suggested to help quenching phenoxy radicals produced upon lignin depolymerization by peroxidases and laccases^65^.

Although laccases have been shown to play an important role in lignin deconstruction^10^, it has been reported that, in contrast to other white-rot fungi, the *P. chrysosporium* genome encodes no laccases^24^. Using the most recent Hidden Markov models in dbCAN, we found two predicted laccases (Table 2; 2762168133 and 2762164443), but none of these were detected in this study.

## Concluding remarks

We have undertaken a proteomics approach to investigate and compare the enzymatic response to growth on biomass, comparing five filamentous fungi, including four Ascomycetes and one white-rot Basidiomycete, grown on three different types of lignocellulosic biomass, pure cellulose and glucose, using agar plate cultures enabling high enrichment of proteins predicted to be secreted. Our data confirm that Ascomycetes mainly degrade carbohydrates, as shown by the lack of detected lignin-active enzymes expressed, despite such enzymes being encoded by the Ascomycete genomes. The white-rot Basidiomycete, on the other hand, expressed a number of oxidoreductases, most of them upregulated only in the presence of lignin.

Although the CAZyme profiles in the secretomes were by and large similar between the fungi, large variations were observed between the fungi and between the lignocellulosic substrates at the level of individual enzymes, or enzyme classes. For the Ascomycete *A. terreus*, this variation, however, was very low, indicating strong and broad co-regulation of CAZyme expression for this fungus. Quantitative proteomics together with heat maps and co-expression network analysis constitute a powerful method for detecting proteins involved in lignocellulose degradation, including well-studied CAZymes that are already exploited industrially, CAZymes with less clear roles in biomass conversion and proteins with other or uncharacterized functions. Proteins such as the unknowns appearing in Fig. 4, the abundantly expressed uncharacterized LPMO from the Basidiomycete *P. chrysosporium* as well as LPMOs and esterases that are only expressed on selected substrates are interesting targets for further research. Certainly, the seemingly central role of the non-CAZy enzymes, such as lactonases, catalases, and lipases deserves further investigation.

## Methods

### Strains, media and culture conditions

*Trichoderma reesei* strain QM6a (ATCC 13631) was obtained from the American Type Culture Collection (ATCC, Manassas, VA, USA). *Aspergillus terreus* strain DSM 1958, *Myceliophthora thermophila* strain DSM 1799 and *Phanerochaete chrysosporium* strain DSM 6909 were obtained from the German Collection of Microorganisms and Cell Cultures GmbH (DSMZ, Braunschweig, Germany). *Neurospora crassa* strain CBS 259.47 was obtained from the Centraalbureau voor Schimmelcultures (CBS, now Westerdijk Fungal Biodiversity Institute, Utrecht, The Netherlands). Spores were stored in 15% glycerol at − 80 °C and the strains were pre-cultured on malt extract agar (MEA; for *P. chrysosporium* and *M. thermophila*), or potato dextrose agar (PDA; for *A. terreus, T. reesei* and *N. crassa*). *T. reesei* and *N. crassa* were cultured at 25 °C, *A. terreus* and *P. chrysosporium* at 35 °C and *M. thermophila* at 45 °C. The agar plates used for growth of the fungi and subsequent extraction of secretomes were prepared as previously described^26^, using modified Mandels medium^66^ where peptone and Tween 80 had been omitted. 10 g/L of either sugarcane bagasse (*Saccharum officinarum*), birch (*Betula pubescens*), spruce (*Picea abies*; Borregaard AS, Sarpsborg, Norway), Sigmacell Cellulose Type 20 (Sigma-Aldrich, St. Louis, MO, USA) or glucose were used as carbon source for growth. Sugarcane bagasse, spruce and birch were milled to pass a sieve of 10 mm (SM2000, Retsch, Haan, Germany) and mixed with liquid medium in a knife mill (GM300, Retsch, Haan, Germany) before use. Agar plates were prepared by sequentially adding two layers of 10 mL medium to petri dishes (Ø = 85 mm) with a sterile Supor 200 0.2-µm membrane (Ø = 47 mm; Pall Life Sciences, Port Washington, NY) in between; see^26^ for details.

### Inoculation and sample preparation

For each fungus, regular agar plates with different carbon sources were inoculated with mycelium from a pre-culture with MEA or PDA and incubated until new mycelium had formed on top, i.e. covering the site of inoculation, large enough to enable sampling; this took longer time for the Basidiomycete than for the Ascomycetes. The cultures were then subsequently used to inoculate plates with matching carbon sources for secretome collection. Inoculation was achieved by using the back of a sterile pipette tip to punch out an agar plug (Ø = 7 mm) and by positioning this plug in the center of the plate, above the membrane. The five fungi were incubated, using three replicates for each carbon source for 3 days. The secretomes were collected as previously described^26^; in brief, the agar was flipped out of the petri dish, exposing the cell-free agar beneath the membrane, and a sterile 50 mL Falcon tube was used to punch out an agar plug under the center of the membrane. Proteins embedded in the agar were reduced by adding 4 µmol dithiothreitol (DTT) per gram of agar. The samples were boiled in two cycles to induce protein desorption and denaturation and further cooled to room temperature (during method optimization, thermal denaturation was validated to have no negative effects on tryptic degradation). The re-solidified agar was then crushed through a syringe to reduce particle size and 1 mL of 100 mM NH_4_HCO_3_ per gram agar was added to get a final concentration of 50 mM NH_4_HCO_3_. 2 µg of Sequencing Grade Modified Trypsin in Trypsin Resuspension Buffer (Promega, Madison, WI, USA) was added to each sample followed by incubation at 37 °C over night. The supernatants, containing tryptic peptides, were collected by freezing and thawing the samples to collapse the gel, followed by centrifugation (16,000×*g*) in order to squeeze out the liquid. Trifluoroacetic acid (TFA) was added to a final concentration of 0.1% (v/v), and the peptides were purified using C18 ZipTips (Merck Millipore, Cork, Ireland) according to the manufacturer’s instructions.

### NanoLC-Orbitrap MS/MS analysis of tryptic peptides

Peptides were analyzed as previously described^67^. In brief, we used a Dionex Ultimate 3000 nanoLC-MS/MS system connected to a Q-Exactive mass spectrometer (both from Thermo Scientific, Bremen, Germany) and equipped with a nano-electrospray ion source. Peptides were loaded onto a trap column (Acclaim PepMap100, C_18_, 5 µm, 100 Å, 300 µm i.d. × 5 mm, Thermo Scientific) and back flushed onto a 50-cm analytical column (Acclaim PepMap RSLC C_18_, 2 µm, 100 Å, 75 µm ID, Thermo Scientific). Initial column conditions were 96% solution A [0.1% (v/v) formic acid], 4% solution B [80% (v/v) acetonitril, 0.1% (v/v) formic acid]. Peptides were eluted using a gradient over 125 min from 4% to 40% (v/v) solution B at a flow rate of 300 nL/min. The Q-Exactive was operated with DDA (data-dependent acquisition) to switch automatically between orbitrap-MS and higher-energy collisional dissociation (HCD) orbitrap-MS/MS in order to isolate and fragment the 10 most intense peptides at any given time throughout the chromatographic elution. The selected precursor ions were excluded for repeated fragmentation for 20 s. The MS resolutions were set to 70,000 and 35,000 for MS and MS/MS, respectively, and automatic gain control (AGC) target values were set to 50,000 charges and a maximum injection time of 128 ms.

### Bioinformatics and co-expression network inference

Proteomes were downloaded from UniProt for *A. terreus* (proteome UP000007963, 10,417 sequences), *T. reesei* (proteome UP000008984, 9,114 sequences), *M. thermophila* (proteome UP000007322, 9,079 sequences) and *N. crassa* (proteome UP000001805, 10,258 sequences). The proteome for *P. chrysosporium* was downloaded from IMG (Genome ID: 2761201668, 13,602 sequences, gene model Phchr2) (available at https://img.jgi.doe.gov/cgi-bin/w/main.cgi). MS raw files were analyzed using MaxQuant^68^ version 1.6.0.13, and proteins were identified and quantified using the MaxLFQ algorithm^69^. The data were searched against the abovementioned proteomes supplemented with common MS and proteomics contaminants such as human keratin and bovine serum albumin. In addition, reversed sequences of all protein entries were concatenated to the databases for estimation of false discovery rates (FDR). The tolerance levels for matching to the database were 6 ppm for MS and 20 ppm for MS/MS. Trypsin was used as digestion enzyme, and two missed cleavages were allowed. Protein N-terminal acetylation, oxidation of methionines, deamidation of asparagines and glutamines and formation of pyro-glutamic acid at N-terminal glutamines were allowed as variable modifications. The ‘match between runs’ feature of MaxQuant, which enables identification transfer between samples based on accurate mass and retention time^69^, was applied with a match time window of one minute and an alignment time window of 20 min. All identifications were filtered in order to achieve a protein FDR of 1%, and only proteins identified in two of three biological replicates on at least one carbon source were kept for further analysis. Note, however, that this initial filtering, while being rigorous, also include proteins identified in only one biological replicate, given that it was found in at least two biological replicates on another carbon source. This dataset was then used to generate all counts and averaged values used in the tables and figures. Further, in order to allow for comparison of expression levels between fungi, all raw files were normalized to the same scale by log2-transformations followed by subtraction of Tukey’s biweight (a robust measurement of the average abundance). Hierarchical clustering was done using Euclidean distance measure and average linkage. Perseus version 1.6.0.7 were used for data analysis and heat map generation. Repeatability were evaluated by multi-scatter plots which showed Pearson correlations between replicates ranging between R = 0.86 – 0.98 (R = 0.67 for *N. crassa* due to few proteins detected).

In order to predict secretion of proteins, we used a combination of three prediction algorithms. SignalP^70^ version 4.1 (available at https://www.cbs.dtu.dk/services/SignalP/) and Phobius^71^ (available at https://phobius.sbc.su.se/) were used with default parameters for eukaryotic species, while WolfPSort^72^ command line version 0.2 was used with a fungal prediction pattern. A protein was considered secreted if at least two of the three prediction algorithms predicted this. Protein names used throughout the manuscript were assigned by UniProt, while further annotated with CAZy family numbers, including carbohydrate-binding modules, using dbCAN^73^ with CAZy hidden Markov models version 6.0, with peptidase family numbers using MEROPS^74^, as well as with Enzyme Commission (EC) numbers and gene ontology (GO) terms, when available, downloaded from UniProt or IMG.

The proteomics data were used to infer the co-expression networks for each fungus using weighted correlation network analysis (WGCNA)^75^ in R. Missing values were substituted with − 10 (lowest LFQ expression value), and the adjacency matrices were computed using the function *adjacency* from the WGCNA package. Initially, network representations were generated for each fungus on the protein level where each node denoted a protein and two nodes were connected by edges whose weight was the Pearson Correlation Coefficient between the expression profile of the two proteins. These networks were then collapsed into secondary networks where the nodes were enzyme classes and the node size indicated the number of enzymes in the class. The interaction between pairs of enzyme classes was computed as the fraction between the collective strength of the edges shared between the enzyme classes and the maximal strength possible on the co-expression networks. The set of vertices V was divided into subsets S_1_, …, S_i_, …, S_n_ representing enzymatic groups such as LPMO, CDH, and others (vertices representing proteins with multiple annotations were included in multiple subsets). The interaction coefficient C_SiSj_ was computed using the following formula:

1 $${C}_{{S}_{i}{S}_{j}}=\frac{{\sum }_{v\in {s}_{i}}{\sum }_{u\in {s}_{j}}{e}_{vu}}{\left(\left|{S}_{i}\right|\cdot \left|{S}_{j}\right|\right)}$$

where e_*vu*_ is the weighted edge between the vertex *v*, from the *i*th enzymatic class, and *u*, from the *j*th one; and |S_*i*_| is the cardinality of the ith subset. In case *i* = *j* (intra-group relation) both numerator and denominator were adjusted by −|S_*i*_| to remove the correlations of the proteins with themselves. One network was created per fungus, and the five networks were further collapsed into one by computing the average for each entry (entries based on missing values were omitted). The strength of the vertices was computed using the function *strength* from igraph^76^. The network was simplified using its discretized version (intervals 0–100 with function *discretize* form arules^77^) and the marginal likelihood filter^78^ (p-value 0.05). The final network was exported to Cytoscape^79^ for visualization.

## Supplementary information

Supplementary Information 1.

Supplementary Information 2.

Supplementary Information 3.

## Data Availability

The mass spectrometry proteomics data have been deposited to the ProteomeXchange Consortium (https://proteomecentral.proteomexchange.org) via the PRIDE partner repository^80^ with the dataset identifier PXD011166. The R notebook and the data necessary to produce the co-expression network analysis are available at https://github.com/fdelogu/fungi-proteomics-net.git.
