# Supplementary material for: Quantitative comparison of the biomass-degrading enzyme repertoires of five filamentous fungi
Source: Sci Rep. 2020 Nov 20;10:20267. doi: 10.1038/s41598-020-75217-z (PMC7679414; doi:10.1038/s41598-020-75217-z)
Supplement: Supplementary file 1 — Supplementary Information 1. [file 41598_2020_75217_MOESM1_ESM.docx]

# SUPPLEMENTARY MATERIAL

**Table S1-S5: Proteins identified in the secretomes of all five fungi.** The tables show the MaxQuant output for identified proteins and the normalized log2-based LFQ intensities used for quantification of *A. terreus, T. reesei, M. thermophila, N. crassa* and *P. chrysosporium*, respectively. Protein annotations were done using SignalP, Phobius, WolfPSORT, dbCAN, UniProt and InterProScan. PEP: posterior error probability. Note that protein names are from UniProt and may in some cases not reflect the CAZyme annotation.

**Figure S1-S5: Heat map representations of quantitative proteomics data of secretomes of the five fungi**. The figures show the expression patterns of proteins detected in the secretomes of *A. terreus, T. reesei, M. thermophila, N. crassa* and *P. chrysosporium*, respectively. Every row in the heat map represents a protein, and the colors represent protein abundance in each of the fifteen samples (three biological replicates for each of the five substrates, sugarcane bagasse, birch, spruce, cellulose and glucose). The colors in the heat map range from high abundance (white color, normalized log2-based LFQ at 10) to low abundance (black color, normalized log2-based LFQ at -5). The grey color indicates that the protein was not detected. All proteins predicted to be secreted are indicated with a green box. The CAZy annotation, as predicted by dbCAN, is shown for all proteins when available, but note that protein names given by UniProt may in some cases not reflect the CAZyme annotation. The figures were made with Perseus v1.6.0.7 and Inkscape v0.48.4 (https://inkscape.org/). **Table S1-S5** shows the data used for generating these figures.

**Figure S6: Heat map representations of selected non-CAZy proteins**. The figure shows the expression patterns of non-CAZy proteins that are predicted to be secreted and that are not expressed during growth on glucose. Further, only proteins with diverse expression patterns are shown here, while proteins consistently expressed on polymeric substrates are found in **Figure 3**. Every row in the heat map represent a protein, and the colors represent the average protein abundance when grown on five different substrates: Sugarcane bagasse, birch, spruce, cellulose and glucose. The colors in the heat map range from high abundance (white color, normalized log2-based LFQ at 10) to low abundance (black color, normalized log2-based LFQ at -5). The grey color indicates that the protein was not detected. Three clusters were manually highlighted to indicate protein groups with similar expression pattern. The figure was made with Perseus v1.6.0.7 and Inkscape v0.48.4 (<https://inkscape.org/>).
