# Supplementary material for: Quantitative comparison of the biomass-degrading enzyme repertoires of five filamentous fungi
Source: Sci Rep. 2020 Nov 20;10:20267. doi: 10.1038/s41598-020-75217-z (PMC7679414; doi:10.1038/s41598-020-75217-z)
Supplement: Supplementary file 2 — Supplementary Information 2. [file 41598_2020_75217_MOESM2_ESM.pdf]

Quantitative comparison of the biomass-degrading enzyme repertoires of five filamentous fungi

Magnus Ø. Arntzen[1*#], Oskar Bengtsson[1#], Anikó Várnai[1], Francesco Delogu[1], Geir Mathiesen[1], Vincent G. H. Eijsink[1]

[1]Faculty of Chemistry, Biotechnology and Food Science, Norwegian University of Life Sciences (NMBU), Ås, Norway.

Fig S1 - Aspergillus terreus

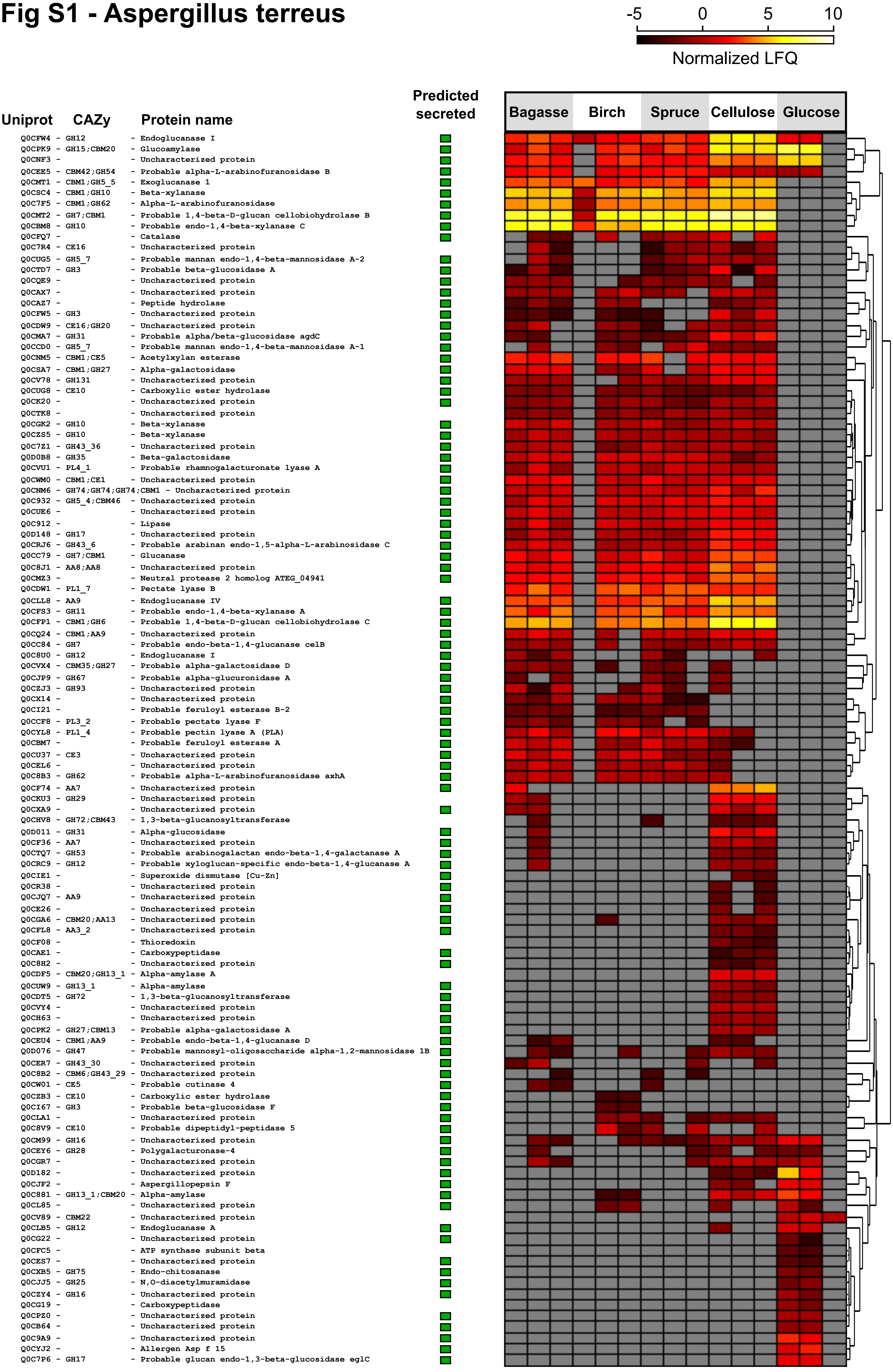

Fig S2 - Trichoderma reesei

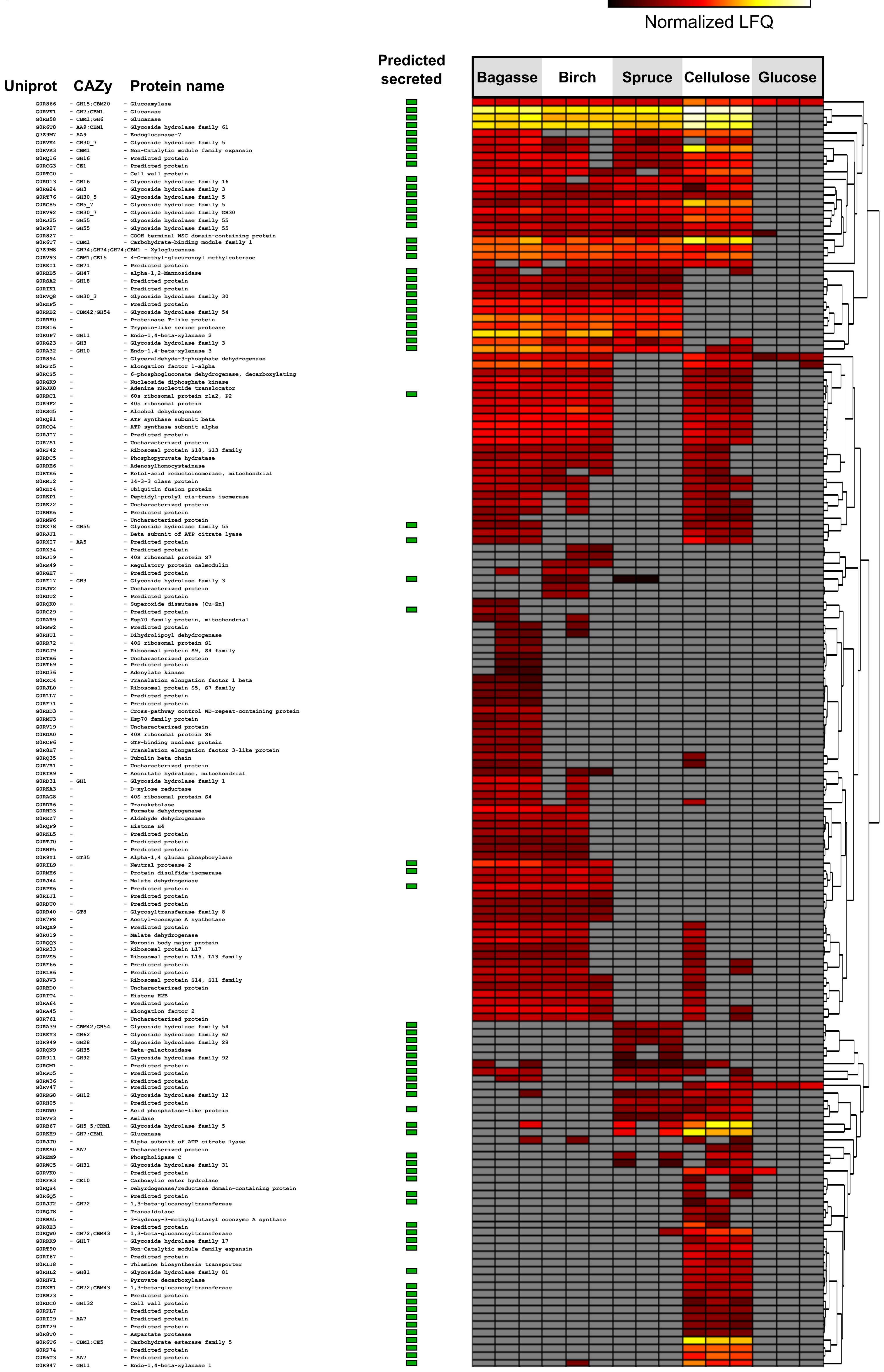

Fig S3 - Myceliophthora thermophila

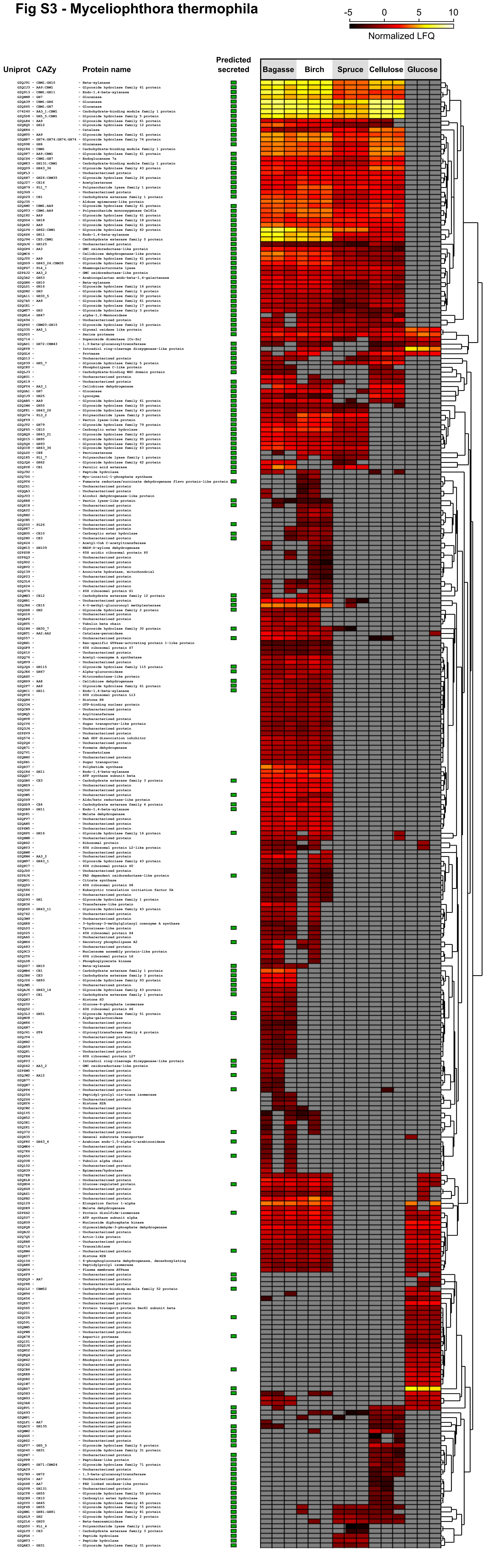

Fig S4 - Neurospora crassa

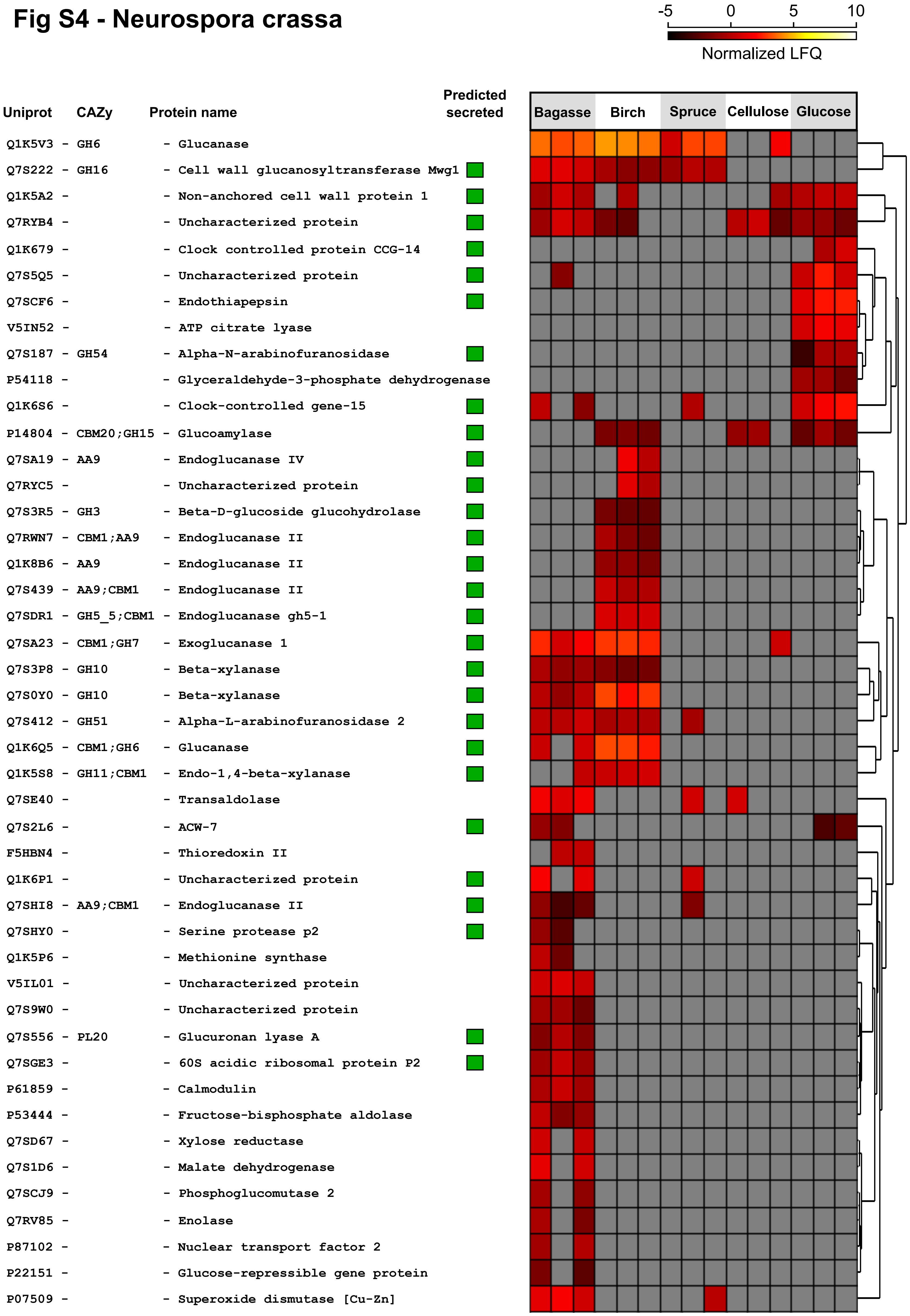

Fig S5 - Phanerochaete chrysosporium

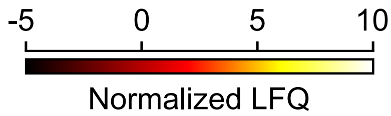

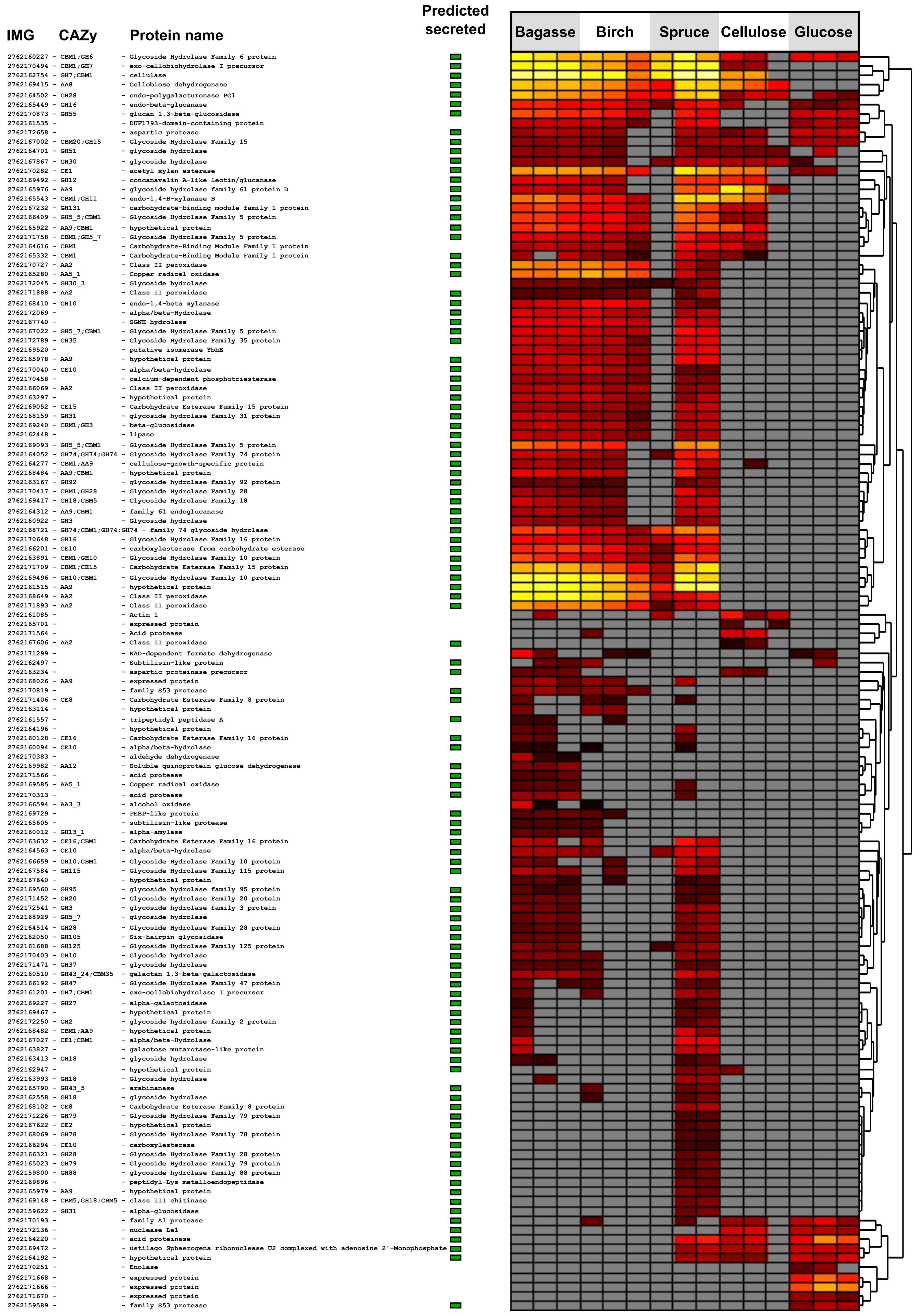

Fig S6

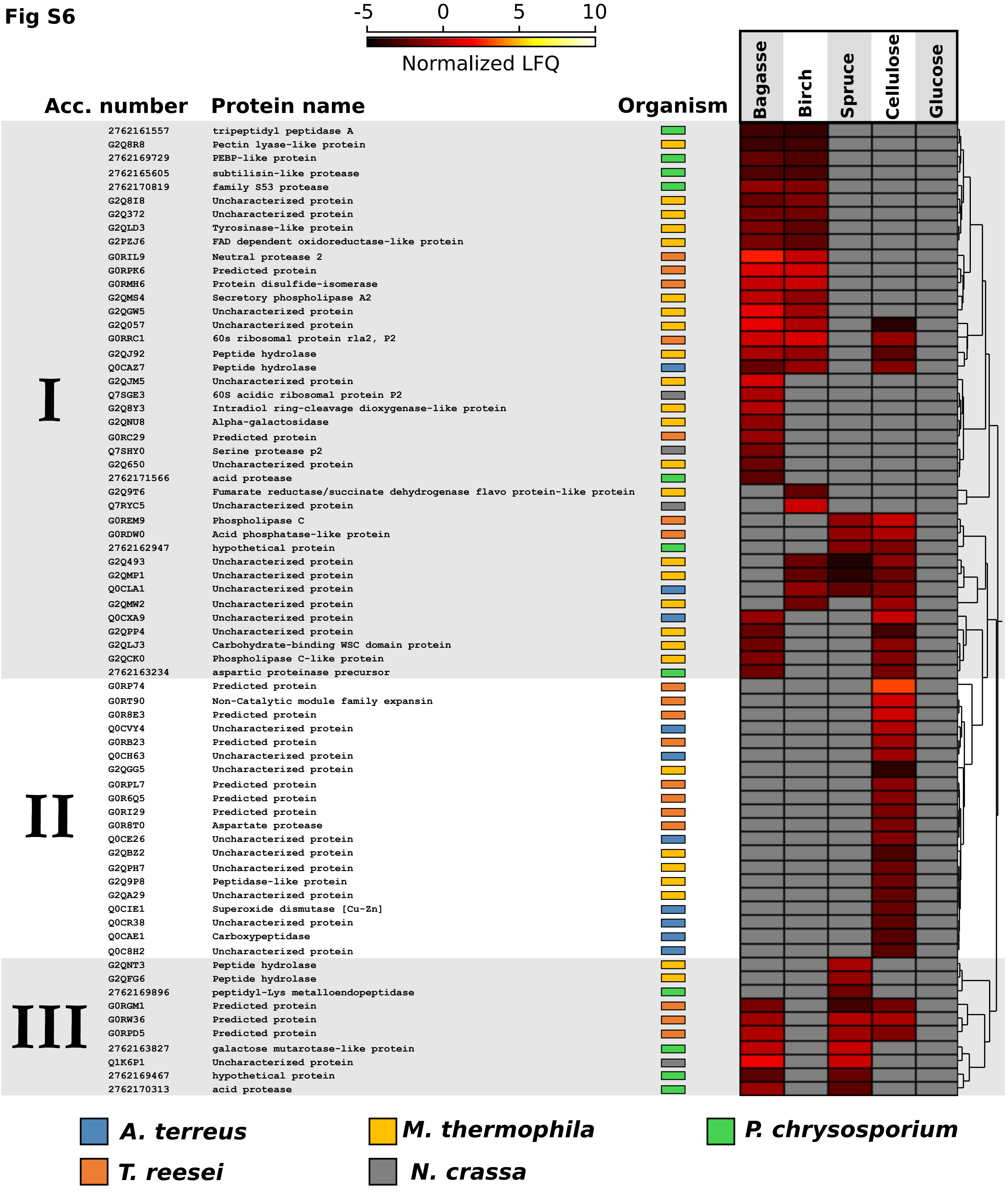

-5
0
5
10
Normalized LFQ
Acc. number
Protein name
Organism
Bagasse
Birch
Spruce
Cellulose
Glucose
I
II
III
2762161557 tripeptidyl peptidase A
G2Q8R8 Pectin lyase-like protein
2762169729 PEBP-like protein
2762165605 subtilisin-like protease
2762170819 family S53 protease
G2Q8I8 Uncharacterized protein
G2Q372 Uncharacterized protein
G2QLD3 Tyrosinase-like protein
G2PZJ6 FAD dependent oxidoreductase-like protein
G0RIL9 Neutral protease 2
G0RPK6 Predicted protein
G0RMH6 Protein disulfide-isomerase
G2QMS4 Secretory phospholipase A2
G2QGW5 Uncharacterized protein
G2Q057 Uncharacterized protein
G0RRC1 60s ribosomal protein rla2, P2
G2QJ92 Peptide hydrolase
Q0CAZ7 Peptide hydrolase
G2QJM5 Uncharacterized protein
Q7SGE3 60S acidic ribosomal protein P2
G2Q8Y3 Intradiol ring-cleavage dioxygenase-like protein
G2QNU8 Alpha-galactosidase
G0RC29 Predicted protein
Q7SHY0 Serine protease p2
G2Q650 Uncharacterized protein
2762171566 acid protease
G2Q9T6 Fumarate reductase/succinate dehydrogenase flavo protein-like protein
Q7RYC5 Uncharacterized protein
G0REM9 Phospholipase C
G0RDW0 Acid phosphatase-like protein
2762162947 hypothetical protein
G2Q493 Uncharacterized protein
G2QMP1 Uncharacterized protein
Q0CLA1 Uncharacterized protein
G2QMW2 Uncharacterized protein
Q0CXA9 Uncharacterized protein
G2QPP4 Uncharacterized protein
G2QLJ3 Carbohydrate-binding WSC domain protein
G2QCK0 Phospholipase C-like protein
2762163234 aspartic proteinase precursor
G0RP74 Predicted protein
G0RT90 Non-Catalytic module family expansin
G0R8E3 Predicted protein
Q0CVY4 Uncharacterized protein
G0RB23 Predicted protein
Q0CH63 Uncharacterized protein
G2QGG5 Uncharacterized protein
G0RPL7 Predicted protein
G0R6Q5 Predicted protein
G0RI29 Predicted protein
G0R8T0 Aspartate protease
Q0CE26 Uncharacterized protein
G2QBZ2 Uncharacterized protein
G2QPH7 Uncharacterized protein
G2Q9P8 Peptidase-like protein
G2QA29 Uncharacterized protein
Q0CIE1 Superoxide dismutase [Cu-Zn]
Q0CR38 Uncharacterized protein
Q0CAE1 Carboxypeptidase
Q0C8H2 Uncharacterized protein
G2QNT3 Peptide hydrolase
G2QFG6 Peptide hydrolase
2762169896 peptidyl-Lys metalloendopeptidase
G0RGM1 Predicted protein
G0RW36 Predicted protein
G0RPD5 Predicted protein
2762163827 galactose mutarotase-like protein
Q1K6P1 Uncharacterized protein
2762169467 hypothetical protein
2762170313 acid protease
A. terreus
M. thermophila
P. chrysosporium
T. reesei
N. crassa
